# The Role of Omega-3 Fatty Acids in Reverse Cholesterol Transport: A Review

**DOI:** 10.3390/nu9101099

**Published:** 2017-10-06

**Authors:** Alex Pizzini, Lukas Lunger, Egon Demetz, Richard Hilbe, Guenter Weiss, Christoph Ebenbichler, Ivan Tancevski

**Affiliations:** 1 Department of Internal Medicine II, Infectious Diseases, Pneumology, Rheumatology, Medical University of Innsbruck, 6020 Innsbruck, Austria; Alex.Pizzini@i-med.ac.at (A.P.); Egon.Demetz@i-med.ac.at (E.D.); Richard.Hilbe@i-med.ac.at (R.H.); Guenter.Weiss@i-med.ac.at (G.W.); 2 Department of Internal Medicine I, Gastroenterology, Hepatology, Endocrinology and Metabolism, Medical University of Innsbruck, 6020 Innsbruck, Austria; Lukas.Lunger@i-med.ac.at (L.L.); Christoph.Ebenbichler@i-med.ac.at (C.E.)

**Keywords:** omega 3, PUFA, *n*-3 PUFA, RCT, Reverse Cholesterol Transport

## Abstract

The beneficial effects of omega-3 polyunsaturated fatty acids (*n*-3 PUFAs) on cardiovascular disease have been studied extensively. However, it remains unclear to what extent *n*-3 PUFAs may impact Reverse Cholesterol Transport (RCT). RCT describes a mechanism by which excess cholesterol from peripheral tissues is transported to the liver for hepatobiliary excretion, thereby inhibiting foam cell formation and the development of atherosclerosis. The aim of this review is to summarize the literature and to provide an updated overview of the effects of *n*-3 PUFAs on key players in RCT, including apoliprotein AI (apoA-I), ATP-binding cassette transporter A1 (ABCA1), ABCG1, apoE, scavenger receptor class B type I (SR-BI), cholesteryl ester transfer protein (CETP), low-density lipoprotein receptor (LDLr), cholesterol 7 alpha-hydroxylase (CYP7A1) and ABCG5/G8. Based on current knowledge, we conclude that *n*-3 PUFAs may beneficially affect RCT, mainly by influencing high-density lipoprotein (HDL) remodeling and by promoting hepatobiliary sterol excretion.

## 1. Introduction

**Figure 1:**
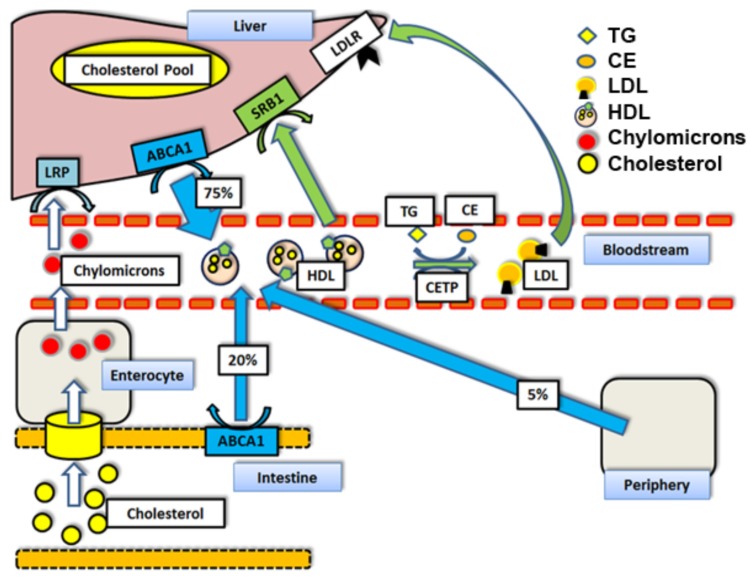
Reverse Cholesterol Transport mechanisms and respective contributions to HDL cholesterol loads by percent [10]: Abbreviations: TG = triglycerides. HDL = high density lipoprotein particle. CETP = cholesteryl ester transfer protein. LDL = low density lipoprotein particle. ABCA1 = ATP binding cassette transporter A1. LDLr = Low density lipoprotein receptor. LRP = LDLr receptor-related protein. SR-BI = scavenger receptor class B type I. CE = cholesteryl ester.

Ischemic heart disease is still the leading cause of death in Western societies [1]. A strong inverse correlation between plasma concentrations of high-density lipoprotein cholesterol (HDL-C) and the incidence of atherosclerotic-driven cardiovascular disease (CVD) has been shown previously [2,3,4]. This has led to the hypothesis that interventions aimed at increasing HDL-C levels might positively influence the risk of CVD [5]. However, data on the efficacy of isolated HDL-C enhancing therapies are scarce and their effects remain controversial [6,7,8,9]. A main atheroprotective function of HDL particles is believed to be translated by the promotion of so-called “Reverse Cholesterol Transport” (RCT). This process describes the clearing pathways of peripheral, subendothelial macrophage- and fibroblast-derived cholesterol, either directly via HDL (hepatic uptake via scavenger receptor B-I, SR-BI), or indirectly by shifting cholesterol from HDL particles to apoB-containing lipoproteins for subsequent uptake into hepatocytes, via low-density lipoprotein receptors (LDLr) (Figure 1) [5,9].

Omega-3 polyunsaturated fatty acids (*n*-3 PUFAs) may beneficially influence the risk of cardiovascular disease (CVD), especially in the secondary prevention of CVD. Recent findings from randomized controlled trials and meta-analyses led to a class IIa recommendation by the American Heart Association for dietary supplementation with *n*-3 PUFAs in populations at high risk of cardiovascular disease [11]. Several molecular pathways and mechanisms of action affecting CVD risk have been found to be influenced by *n*-3 PUFAs, including the alteration of physical and chemical properties of cellular membranes, direct interaction with, and modulation of, membrane channels and proteins, regulation of gene expression via nuclear receptors and transcription factors, changes in eicosanoid profiles, and the conversion of *n*-3 PUFAs to bioactive metabolites [12]. So far, effects on HDL-C plasma levels have been shown to be only moderate [13,14,15].

**Figure 2:**
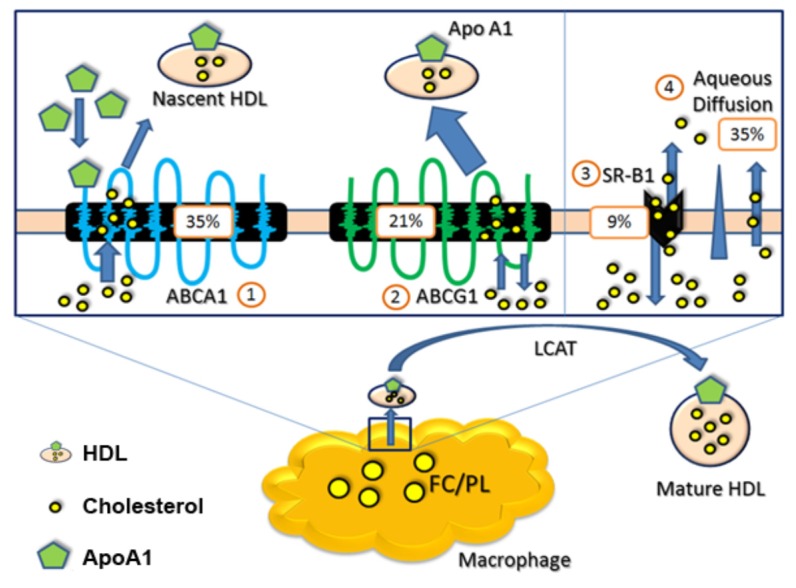
Macrophage cholesterol efflux routes and relative contributions to cholesterol efflux by percent [10]. Abbreviations: ABCA1 = ATP-binding cassette transporter A1, ABCG1 = ATP-binding cassette transporter G1, apo A1 = apolipoprotein A1, FC/PL = free cholesterol/phospholipids, HDL = high density lipoprotein, SR-BI = scavenger receptor class B type I. LCAT = lecithin cholesterol acyl transferase.

Cholesterol metabolism is divided into an exogenous and an endogenous pathway; in the endogenous pathway, cholesterol is synthesized by the liver and extrahepatic tissues, and enters the circulation as a component of lipoproteins, or is secreted into bile. In the exogenous pathway, cholesterol from dietary and biliary sources is absorbed in the intestine and ultimately enters the circulation [16]. Due to the absence of peripheral cholesterol catabolic mechanisms, efficient efflux of intracellular cholesterol and subsequent transport of peripheral cholesterol to the liver is crucial, to avoid the accumulation of cytotoxic cholesterol crystals and the formation of atherosclerotic plaques in arteries [17]. Four cholesterol efflux routes (two active and two passive processes) have been described: ATP-binding cassette transporters A1 (ABCA1) and G1 (ABCG1) (active), aqueous diffusion and SR-BI facilitated cholesterol desorption (passive) (Figure 2) [10,17]. Cholesterol efflux, the first step of RCT, prevents intracellular cholesterol accumulation and has been shown to correlate with protection from atherosclerotic disease [17,18,19]. HDL particles are crucial mediators of cholesterol efflux from lipid-laden macrophages [17,20]. HDL particles comprise a heterogeneous lipoprotein population that differs in size, charge, density and composition [21].

The beneficial effects of omega-3 polyunsaturated fatty acids (*n*-3 PUFAs) on dyslipidemia are well established in literature. It has been found previously that eicosapentaenoic acid (EPA) and docosahexaenoic acid (DHA), the two most important *n*-3 PUFAs in human physiology, may exert their atheroprotective functions by promoting intracellular catabolism of apolipoprotein B-100 containing lipoproteins, suppressing hepatic apoB production, stimulating plasma triglyceride clearance via lipoprotein lipase (LPL), increasing the very low density lipoprotein (VLDL) to LDL conversion rate, reducing LDL synthesis and attenuating postprandial lipemia [22,23,24,25]. It appears likely that *n*-3 PUFAs may mediate these effects, predominately by inhibiting sterol regulatory element-binding protein-1 (SREBP-1) mediated pathways, including the activation of the nuclear transcription factors, Hepatocyte Nuclear Factor-4 Alpha (HNF4A), Farnesoid X Receptor, Liver X Receptor (LXR), and Peroxisome Proliferator-activated Receptors (PPARs) [26].

Even though previous literature investigating the effect of *n*-3 PUFAs and their effects on cholesterol efflux routes and RCT is still ambiguous, it has been clearly shown that *n*-3 PUFAs promote the RCT mechanism. Using an established RCT model, in which (^3^H)-labeled macrophages are injected into the peritoneal cavity of animals—where the appearance of a tracer in plasma and feces is monitored—different laboratories have demonstrated that *n*-3 PUFAs promote macrophage-to-feces RCT in rodents [27,28,29]. In human macrophages, in turn, *n*-3 PUFAs have been shown to significantly increase cholesterol efflux from macrophages [30].

This review aims to summarize current knowledge on the effects of *n*-3 PUFAs on RCT and related pathways.

### 1.1. Apolipoprotein AI (apoA-I), Paraoxonase-1 (PON1), and *n*-3 PUFAs

ApoA-I is the principal protein component of HDL particles. It mediates HDL maturation by promoting ABCA1-mediated cholesterol efflux from cells, and by stimulating enzymatic cholesterol esterification via lecithin cholesterol acyl transferase (LCAT) [10,17,31]. Plasma LCAT activity was increased in hamsters fed a high-fat diet enriched with *n*-3 PUFAs (*n*-3 PUFA oil mixture), which was associated with an increased macrophage-to-feces RCT [29]. Moreover, *n*-3 PUFAs (fish oil, perilla oil, soybean oil) were found to increase hepatic protein expression of apoA-I in obese insulin resistant rats, resulting in significantly increased apoA-I plasma levels [32]. However, clear data demonstrating increased cholesterol efflux from macrophages through induction of apoA-I are lacking.

**Figure 3:**
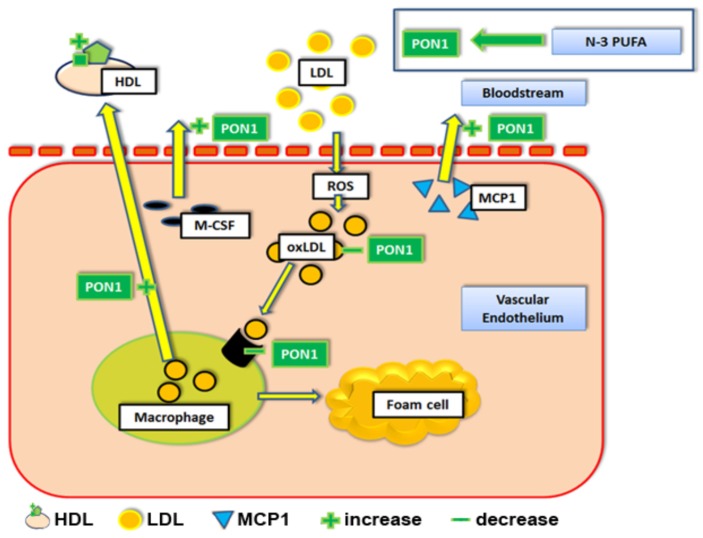
The effects of *n*-3 PUFAs on paraoxonase 1 and cholesterol efflux. Abbreviations: HDL = high density lipoprotein particle. LDL = low density lipoprotein. PON1 = paraoxonase 1. MCP1 = Monocyte chemotactic protein 1. ROS = reactive oxygen species. M-CSF = macrophage colony stimulating factor. oxLDL = oxidized LDL. *n*-3 PUFA = Omega 3 polyunsaturated fatty acid.

Interestingly, induction of apoA-I expression by *n*-3 PUFAs (EPA) may also critically influence the anti-oxidative effects of HDLs [33,34]. ApoA-I is known to stabilize the enzymatic activity of paraoxonase-1 (PON1) [35,36] which associates with HDL particles. PON1, in turn, prevents oxidative modification of LDLs, detoxifies oxidized LDLs (oxLDL), inhibits uptake of oxLDLs by macrophages, and reduces macrophage oxidative stress. Overall, PON1 transgenic *apoE^−/−^* mice have been found to have dramatically reduced levels of pro-inflammatory cytokines within aortic tissues compared to solely *apoE^−/−^* mice (e.g. macrophage colony stimulation factor, M-CSF and in monocyte chemotactic protein, MCP1, Figure 3) [36,37,38], which may result from their antioxidative effects in locally accumulating cells of myeloid origin. Notably, it was also suggested that EPA may enhance the antioxidant qualities of HDLs by activating HDL-associated PON1, independently of apoA-I [34,39]. Finally, PON1 has also been shown to increase the cholesterol efflux capacity of macrophages; both human Tamm-Horsfall protein 1 (THP-1) macrophages as well as murine J774 macrophages displayed a dose-dependent increase in cholesterol efflux when treated with increasing concentrations of PON1 [40].

Taken together, *n*-3 PUFAs seem not to exert direct effects on the lipoprotein cholesterol content, but rather modify HDL composition and function, resulting in increased HDL maturation and PON1-dependent antioxidative effects.

### 1.2. ATP-Binding Cassette Transporter A1 (ABCA1) and *n*-3 PUFAs

ABCA1 plays a key role in the efflux of phospholipids and free cholesterol from cells to lipid-free apoA-I [31,41], leading to the generation of discoidal nascent HDL particles [17,42]. In a subsequent step, LCAT facilitates the condensation of cholesterol into the lipoprotein core via esterification, yielding spherical mature HDL particles [10,43,44]. Mature HDL particles require supplementary, non-ABCA1 mediated lipidation, either via ABCG1, aqueous diffusion or SR-BI mediated pathways [10,17,45].

*n*-3 PUFAs appear to have diverging effects on macrophage-specific cholesterol efflux in mice and in humans. In vitro studies showed that EPA impairs ABCA1 mediated cholesterol efflux in both human and murine macrophages, whereas DHA stimulates ABCA1 dependent efflux in murine macrophages [46]. Chadli et al. as well as Nishimoto et al. reported *n*-3 PUFAs (*n*-3 PUFA oil mixture; EPA) to stimulate RCT, which was reflected by increased fecal sterol excretion [27,29]. However, the authors failed to prove a stimulation of HDL-mediated cholesterol efflux from macrophages. In summary, *n*-3 PUFAs appear not to promote ABCA1/apoA-I mediated cholesterol efflux from macrophages [29,47].

### 1.3. ATP-Binding Cassette Transporter G1 (ABCG1) and *n*-3 PUFAs

Pertaining to the family of ATP-binding cassette transporters, ABCG1 is an integral membrane protein which actively pumps intracellular cholesterol to the outer plasma membrane, leaving it to freely desorb into the extracellular milieu [17]. In contrast to ABCA1, ABCG1 does not enhance the transport of cholesterol and phospholipids to lipid-free apoA-I, rather it mediates the efflux of cellular cholesterol to mature HDL [48,49].

It appears that major emphasis has been put on investigating ABCG1 in in vitro gene expression following a *n*-3 PUFA challenge. Available studies suggest that EPA treatment may reduce ABCG1 expression in human THP-1 monocytic cells; however, this does not occur in fully differentiated macrophages [50]. Further in vitro studies by Uehara et al. corroborated these findings and suggested EPA as a suppressor of the transcription and protein expression of ABCG1 and ABCA1 in murine RAW 264.7 macrophages [47].

ABCA1 and ABCG1 are not only expressed in macrophages, but also in liver cells, where they critically influence HDL synthesis and maturation. As mentioned above, *n*-3 PUFAs have been found to increase macrophage-to-feces RCT in hamsters, which on one hand could be traced back to increased hepatobiliary flux of bile acids and cholesterol, on the other hand appears to be due to an enhanced cholesterol acceptor capacity of HDL [29].

### 1.4. Apolipoprotein E (apoE) and *n*-3 PUFAs

ApoE is a further important apolipoprotein found in HDL particles, and its role in RCT is well established—apoE enhances cholesterol efflux via ABCA1 in macrophages and delivers HDL cholesterol to the liver through interactions with SR-BI and LDLr. It also facilitates the maturation of large HDL particles through LCAT [10]. Most available studies on the interplay between *n*-3 PUFAs and apoE relate to Alzheimer’s disease; previous findings suggest apoE4 polymorphisms to be a genetic risk factor for late onset Alzheimer’s disease [51], and epidemiological studies have postulated a clinical benefit of dietary supplementation with *n*-3 PUFAs in patients with dementia [51,52,53]. Studies investigating the direct effects of *n*-3 PUFAs on apoE expression and function are scarce since the most commonly used atherosclerosis model is the apoE knockout mouse [54].

Yet, in obese rats fed a mixture of *n*-3 and *n*-6 PUFAs (blend of safflower and soybean oil), an increase in apoE within HDL particles has been observed [55]. However, the study did not investigate the effects of isolated *n*-3 PUFA supplementation on apoE plasma levels. A recent study in healthy volunteers with an *n*-3 PUFA (EPA:DHA ratio = 0.54) enriched diet showed a significant increase in apoE within the LDL fraction and in a subgroup with reduced triglyceride levels, an increase in total plasma apoE concentrations [56]. Interestingly, genetic polymorphisms of apoE seem to impact the metabolism and bioavailability of supplemented EPA and DHA [57,58,59].

### 1.5. Scavenger Receptor Class B Type I (SR-BI) and *n*-3 PUFAs

The scavenger receptor class B type I (SR-BI) is a transporter which, in contrast to ABCA1 and ABCG1, enables passive bidirectional flux of cholesterol between cells and HDL particles [10].

To understand the impact of *n*-3 PUFAs on SR-BI, it is crucial to mention that this pathway is involved not only in the desorption of peripheral cholesterol to HDL particles, but also in the hepatic clearance of HDL derived cholesterol [10]. Therefore, in peripheral macrophages, the relative contribution of SR-BI mediated cellular cholesterol efflux has been controversially discussed. Some animal studies have suggested a significant efflux function of peripheral SR-BI [60,61], whereas other results from cell cultures and animal models are ambiguous [62,63,64,65]. It therefore appears that peripheral SR-BI mediated cholesterol efflux may play a minor role, as compared to clearance via ABCG1 and ABCA1 [64].

On the other hand, the major importance of SR-BI lies in its ability to clear HDL cholesterol via hepatic cells, an important step in RCT [66]. Little is known about PUFAs and their effects on SR-BI; a study in genetically obese mutant rats fed a combination of *n*-3 and *n*-6 PUFAs (blend of safflower and soybean oil) showed an increase in hepatic SR-BI expression [55]. ApoE knock out mice, fed different ratios of *n*-3/*n*-6 PUFAs (*n*3-PUFA derived from perilla seed oil containing mostly alpha-linolenic acid (ALA), and minimal amounts of EPA and DHA; safflower oil containing 83% Linoleic acid (LA) was used as the primary source of n-6 fatty acids) displayed reduced hepatic SR-BI mRNA levels when fed a relatively low *n*-3/*n*-6 PUFA ratio [67]. In high *n*-3/*n*-6 PUFA ratio fed mice, hepatic SR-BI mRNA as well as HDL particle plasma levels increased, while no effect on atherogenesis was observed. Chadli et al. analysed the impact of a high-fat diet, supplemented with *n*-3 PUFAs in hamsters, an animal species expressing Cholesteryl Ester Transfer Protein (CETP) [29]. This study revealed, as mentioned above, an increase in RCT following *n*-3 PUFA treatment, probably due to increased expression of the main hepatic genes involved in RCT, including SR-BI [29].

A recent in vitro analysis by Mashurabad et al. showed that EPA leads to a downregulation in SR-BI via PPARα-dependent mechanisms in enterocytes (Caco-2/TC7 cell line), suggesting that the implications of *n*-3 PUFAs on the translation of RCT modifying proteins do not only occur in hepatocytes [68].

In summary, the available literature shows an effect of *n*-3 PUFAs on SR-BI expression in hepatocytes, which may translate into elevated plasma HDL cholesterol clearance as part of the RCT. No data is available about *n*-3 PUFA and cholesterol efflux, which is driven through SR-BI from peripheral macrophages.

### 1.6. Cholesterol Ester Transfer Protein (CETP) and *n*-3 PUFAs

CETP is a plasma glycoprotein secreted by the liver which enables the exchange of cholesteryl esters between HDL and other lipoproteins, such as LDL, thereby promoting hepatic clearance of macrophage-derived cholesterol, via an indirect pathway by hepatic LDLr.

CETP transgenic mice treated with fish oil containing *n*-3 PUFAs showed significant increases in CETP plasma levels and activity, as well as elevated CETP mRNA expression in the liver [69]. The authors hypothesized a PPARα-related induction of CETP transcription through binding of *n*-3 PUFAs to PPARα response elements in the CETP gene [70], since PUFAs have been described as natural ligands for PPARα [71]. In a recent study in hamsters fed a high-fat diet and administered EPA or DHA, distinct results for the CETP-LDL cholesterol pathways were observed [72]. It was found that DHA, in contrast to EPA, led to a significant increase in LDL cholesterol concentrations, an increase in plasma CETP activity as well as elevated CETP mRNA expression in adipose tissue. The effect of EPA on the same parameters, however, was less prominent and suggests that the impact of *n*-3 PUFAs on CETP and RCT may depend on the type of *n*-3 PUFA administered [72]. These findings underline the importance of distinct analyses of different PUFA subclasses, as they may exert differential effects on cholesterol metabolism [73].

### 1.7. LDL-Receptor (LDLr) and *n*-3 PUFAs

Hepatic LDLr mediated uptake of circulating LDL cholesterol constitutes a final part of RCT. However, the effects of *n*-3 PUFAs on this last step of RCT are not entirely clear. Animal studies in LDLr deficient mice suggest that the beneficial effect of *n*-3 PUFAs (EPA, DHA and docosapentaenoic acid *n*-3) on serum lipoprotein profiles and aortic plaque formation may be independent of LDLr [74]. Also, another study by Vasandani underlined the hypothesis that *n*-3 PUFAs (EPA:DHA ratio = 1.48) may decrease the plasma concentration of apoB-containing lipoproteins, independent of LDLr or lipoprotein receptor-related protein mediated pathways [75].

It was previously shown that *n*-3 PUFAs promote post-ER presecretory proteolysis (PERPP)/autophagy, which degrades apoB100, thereby critically reducing lipoprotein secretion from the liver. *n*-3 PUFA induced autophagy was shown to be dependent on lipid peroxidation, which could be blocked by antioxidants, including vitamin E [76,77]. The manipulation of oxidation in humans for the purpose of preventing CVD has received substantial attention and effort. Administration of antioxidants, such as vitamin E, has generally lessened arterial lesions in animal models of atherosclerosis but has not resulted in consistent benefits [78] and has even caused occasional harm in humans [79,80]. Results by Fisher and co-workers have implied that the potential benefits of antioxidants on human arterial lesion development might be counterbalanced by potentially harmful alterations of apoB100 metabolism [76], which raises the question of whether similar effects may also hamper the hepatic secretion of proteins involved in RCT.

### 1.8. Cholesterol 7alpha-Hydroxylase (CYP7A1), ABCG5/G8, NPC1L1 and *n*-3 PUFAs

The last steps of RCT comprise hepatobiliary excretion of neutral sterols via ABCG5/G8, or excretion of acidic sterols after conversion of cholesterol into bile acids via cholesterol 7alpha-hydroxylase 1 (CYP7A1). Bérard et al. showed that dietary supplementation with *n*-3 PUFAs (EPA:DHA ratio = 1.46) induces CYP7A1 transcription through the D-site binding protein and LXR in murine hepatocytes [81]. *n*-3 PUFAs appear to increase bile acid synthesis by inducing hepatic CYP7A1 expression and to enhance hepatobiliary excretion of neutral sterols via ABCG5 in hamsters, which together should promote macrophage-to-feces RCT [29]. Increased hepatobiliary excretion of neutral sterols has also been observed in experiments in C57BL/6 mice fed a diet supplemented with fish oil, most likely through hepatic upregulation of ABCG5/G8 and intestinal downregulation of Niemann-Pick C1-like protein 1 (NPC1L1), which drives intestinal sterol reabsorption under physiological conditions [27].

Together with the liver, the intestine serves as a homeostatic organ in cholesterol metabolism. Recent evidence has substantiated the pivotal role of intestinal RCT. This mechanism has been described as trans-intestinal excretion of plasma-derived cholesterol (TICE) [82]. Located at the brush border membrane of the small intestine, ABCG5/G8 is the main contributor to TICE; ABCB1a/b might play a role as an additional route. Whereas no influence of *n*-3-PUFAs on intestinal ABCB1a/b has been investigated so far, there is evidence that DHA and EPA may affect intestinal cholesterol absorption by downregulating NPC1L1 [83], which, in turn, may critically affect ABCG5/G8 mediated trans-intestinal cholesterol excretion and thereby RCT.

## 2. Conclusions

*n*-3 PUFAs have a positive impact on atherosclerosis and CVD, a major concern of today’s health care systems. It is important to emphasize that the positive effects of *n*-3 PUFAs on CVD are thought to be mediated by diverse mechanisms, including the alteration of physical and chemical properties of cellular membranes, direct interaction with, and modulation of, membrane channels and proteins, regulation of gene expression via nuclear receptors and transcription factors, changes in eicosanoid profiles, and conversion of *n*-3 PUFAs to bioactive metabolites, which may promote ischemia-induced myocyte healing.

The aim of this article was to review possible beneficial effects of *n*-3 PUFAs on RCT. After evaluating available literature, it appears likely that this effect is, to a certain degree, mediated by positively influencing RCT on different levels, as illustrated by the stimulation of macrophage-to-feces RCT. However, these studies may not adequately illustrate the complexity of involved mechanisms and steps. Taking a closer look at the pivotal steps of RCT, the available literature suggests that *n*-3 PUFAs might affect several, but not all, involved processes. Interpretation of available studies is difficult and hampered by heterogenous study designs, sample sizes, different PUFA formulations used (*n*-3/n-6 PUFAs, phytosterols and/or single formulations of EPA or DHA etc.), and varying experimental approaches (in vivo versus in vitro). The application of results derived from mouse studies is limited by striking differences between murine and human lipoprotein metabolism, the mouse being an ‘HDL’ animal without CETP in plasma. Available studies in hamsters, which naturally express CETP, may help to better extrapolate observed physiological effects of *n*-3 PUFAs on RCT to the human setting. However, the number of studies in this animal model is limited, warranting further experiments in CETP-expressing, potentially dyslipidemic animal models to verify existing hypotheses on *n*-3 PUFAs and RCT. In summary, *n*-3 PUFAs may not significantly affect the first step of RCT—i.e., ATP-dependent cholesterol efflux mechanisms within atherosclerotic plaque macrophages—rather, they appear to beneficially affect HDL remodeling through LCAT and CETP, facilitating SR-BI and LDLr mediated hepatic uptake of plaque-derived excess cholesterol. In addition, *n*-3 PUFAs were clearly shown to promote hepatobiliary excretion of neutral and of acidic sterols, which is a pivotal final step in RCT.

Gaining further insight into the functionality of RCT may help to determine key populations who may substantially benefit from *n*-3 PUFA supplementation, an easy-to-obtain and interesting dietary supplement, with potentially promising effects in CVD.
